# Supplementary material for: Factor structure of intelligence and divergent thinking subtests: A registered report
Source: PLoS One. 2022 Sep 19;17(9):e0274921. doi: 10.1371/journal.pone.0274921 (PMC9484676; doi:10.1371/journal.pone.0274921)
Supplement: S1 File — (PDF) [file pone.0274921.s001.pdf]

Supplemental File for

Factor Structure of Intelligence and Divergent Thinking Subtests: A Registered Report

This file reports the model fit statistics of the supplemental confirmatory factor analysis models. These models are identical to the models reported in the article, except the TTCT-F creative strengths checklist adjusted score has been dropped from analysis.

Table S1

Fit Statistics for CFA Models

Model	χ^2	df	CFI	TLI	RMSEA [90% CI]	SRMR	AIC	BIC
S1a	1,314.074	66	.891	.859	.079 [.067, .091]	.075	18,334.513	18,492.728
S1b	186.967	51	.891	.859	.079 [.067, .091]	.075	18,334.513	18,492.728
S2a	521.873	53	.624	.532	.144 [.133, .155]	.114	18,665.720	18,815.521
S2b	—	—	—	—	—	—	—	—
S3a	—	—	—	—	—	—	—	—
S3b	—	—	—	—	—	—	—	—
S4a	—	—	—	—	—	—	—	—
S4b	—	—	—	—	—	—	—	—
S5	1,009.100	55	.236	.083	.202 [.191, .213]	.150	19,148.647	19,179.566

Note. Models S2b through S4b did not converge.

When interpreting these models, it is important to be aware of anomalies in some of the models:

- In Model S1a, the adjusted TTCT-F elaboration score has a negative residual variance.
- In Model S1b, the adjusted TTCT-F elaboration score has a negative residual variance. Additionally, the second-order factor loadings on the *g* factor could not be estimated.
- In Model S2a, the adjusted TTCT-F elaboration score has a negative residual variance.
